# Gastric Duplication Cyst in Association with Duodenal Atresia in a Neonate

**Published:** 2016-01-01

**Authors:** Javad Ghoroubi, Alireza Mirshemirani, Fatollah Roshanzamir, Sajad Razavi, Mehdi Sarafi

**Affiliations:** 1 Pediatric Surgery Research Center, Shahid Beheshti University of Medial Sciences Tehran Iran; 2 Pediatric Anesthesiology Department, Shahid Beheshti University of Medial Sciences Tehran Iran

**Keywords:** Gastric duplication, Duodenal atresia, Neonate

## Abstract

Concurrence of duodenal atresia and gastric duplication cyst is extremely rare entity. We report a 6-day-old female neonate who presented with neonatal intestinal obstruction. X-ray abdomen showed double bubble sign. At laparotomy, a huge cystic structure attached to greater curvature of the stomach along with duodenal atresia of second part of duodenum was found. The cystic structure was excised and duodeno-duodenostomy performed. Histopathology report confirmed it gastric duplication cyst.

## CASE REPORT

A 6-day-old female neonate developed signs of neonatal intestinal obstruction. She was a product of twin pregnancy with gestational age of 35 weeks. At birth, she underwent CPR in delivery room, and after intubation received one dose of surfactant. The other twin was normal. On the 4th day she was extubated but very next day developed bilious vomiting and was referred to our center. Thoraco-abdominal x-ray revealed double bubble sign in favor of duodenal obstruction (Fig. 1). Cardiology consultation revealed multiple cardiac anomalies. She underwent CT angiography later in the course of management which confirmed coarctation of the aorta. At laparotomy a distended stomach and huge cystic structure (communicating) attached to the greater curvature of stomach was found (Fig. 2). Further exploration revealed duodenal atresia type I with annular pancreas at 2nd part of the duodenum. The cyst excised and gastric wall repaired. Duodeno-duodenostomy was then carried out. Postoperative recovery was uneventful. The infant is doing well at six months follow-up.

**Figure:**
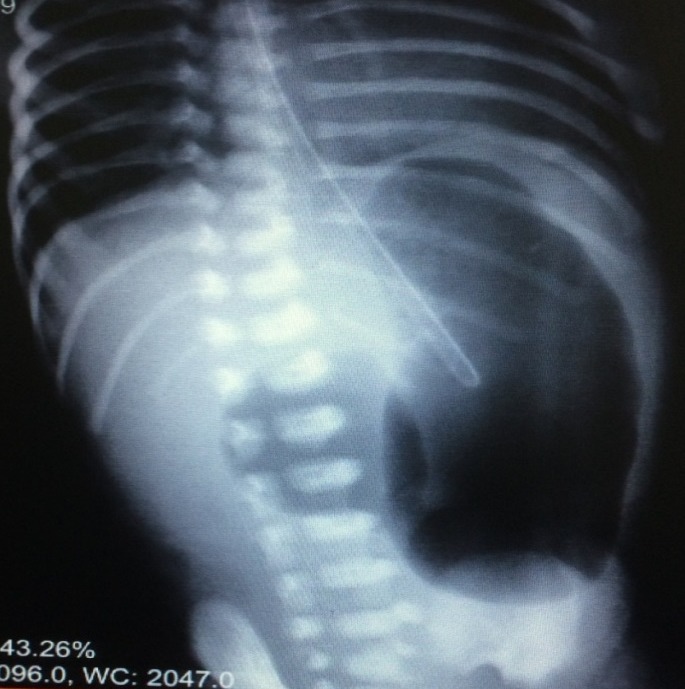
Figure 1:Double bubble sign.

**Figure:**
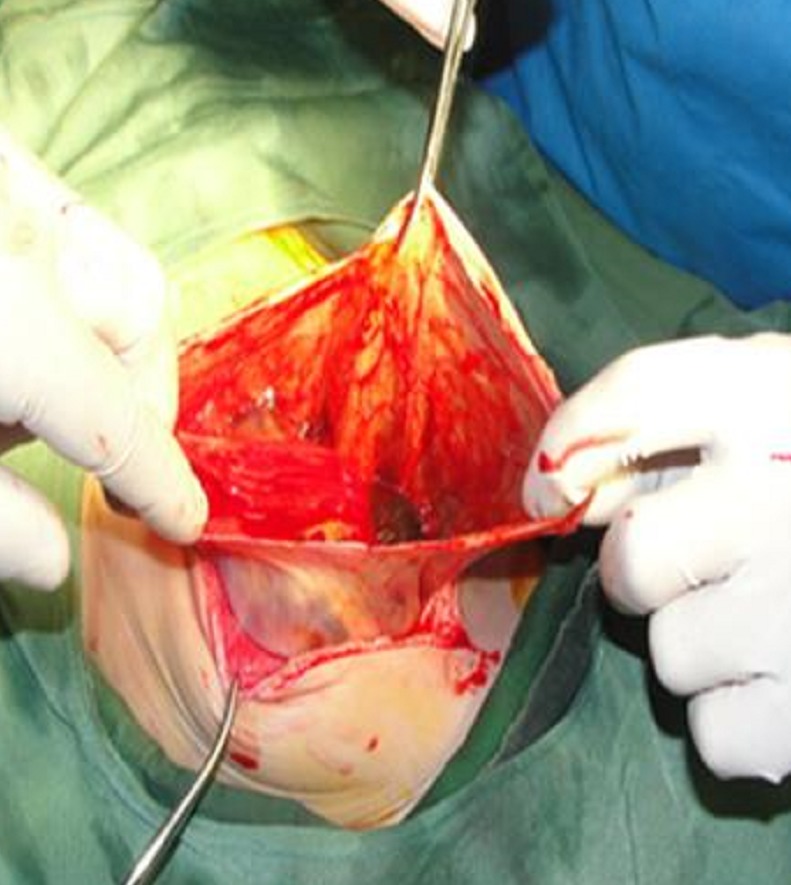
Figure 2:Gastric cystic lesion after opening.

## DISCUSSION

Gastric duplications (GD) are the rare alimentary tract duplications.[1] GDs mostly present with vague abdominal pain, vomiting, and occasionally a palpable abdominal mass. These may remain asymptomatic or present with complications.[2] GDs have occasionally been reported with intestinal atresia.[3,4] Few case reports of GIT duplications and atresia of the adjacent part of normal alimentary tract are available on literature search.[3-5] In our case gastric duplication was associated with duodenal atresia and annular pancreas. Presence of cyst may have some role in vascular accidents which might have resulted in atresia of the adjacent part. Surgery is the mainstay of treatment.[2] In our case, the cyst was huge in size and communicating with stomach. Histopathology confirmed gastric mucosa in our case.

In conclusion, concurrence of gastric duplication cyst with duodenal atresia is a rare event. Presence of duplication cyst may have some role in genesis of duodenal atresia in the index case.

## Footnotes

**Source of Support:** Nil

**Conflict of Interest:** None declared

